# Genome-wide and molecular evolution analysis of the subtilase gene family in *Vitis vinifera*

**DOI:** 10.1186/1471-2164-15-1116

**Published:** 2014-12-16

**Authors:** Jun Cao, Xi Han, Ticao Zhang, Yongping Yang, Jinling Huang, Xiangyang Hu

**Affiliations:** Key Laboratory for Plant Diversity and Biogeography of East Asia, Kunming Institute of Botany, Chinese Academy of Sciences, Kunming, China; The Germplasm Bank of Wild Species, Kunming Institute of Botany, Chinese Academy of Sciences, Kunming, China; Institute of Life Sciences, Jiangsu University, Zhenjiang, Jiangsu 212013 China; University of Chinese Academy of Sciences, Beijing, China; Department of Biology, East Carolina University, Greenville, NC 27858 USA

**Keywords:** *Vitis vinifera*, Subtilase, Gene family, Evolution, Positive selection, Differential expression

## Abstract

**Background:**

*Vitis vinifera* (grape) is one of the most economically significant fruit crops in the world. The availability of the recently released grape genome sequence offers an opportunity to identify and analyze some important gene families in this species. Subtilases are a group of subtilisin-like serine proteases that are involved in many biological processes in plants. However, no comprehensive study incorporating phylogeny, chromosomal location and gene duplication, gene organization, functional divergence, selective pressure and expression profiling has been reported so far for the grape.

**Results:**

In the present study, a comprehensive analysis of the subtilase gene family in *V. vinifera* was performed. Eighty subtilase genes were identified. Phylogenetic analyses indicated that these subtilase genes comprised eight groups. The gene organization is considerably conserved among the groups. Distribution of the subtilase genes is non-random across the chromosomes. A high proportion of these genes are preferentially clustered, indicating that tandem duplications may have contributed significantly to the expansion of the subtilase gene family. Analyses of divergence and adaptive evolution show that while purifying selection may have been the main force driving the evolution of grape subtilases, some of the critical sites responsible for the divergence may have been under positive selection. Further analyses of real-time PCR data suggested that many subtilase genes might be important in the stress response and functional development of plants.

**Conclusions:**

Tandem duplications as well as purifying and positive selections have contributed to the functional divergence of subtilase genes in *V. vinifera*. The data may contribute to a better understanding of the grape subtilase gene family.

**Electronic supplementary material:**

The online version of this article (doi:10.1186/1471-2164-15-1116) contains supplementary material, which is available to authorized users.

## Background

Subtilases are a very diverse family of subtilisin-like serine proteases found in all three domains of life (bacteria, archaea and eukaryotes). They are characterized by a catalytic triad of Asp, His, and Ser residues or a conserved catalytic residue Asn in an arrangement shared with subtilisins from *Bacillus* [1–3], all of which are located in the N-terminal domains of the mature enzymes. Most subtilases have a multi-domain structure, comprising a signal peptide, a pro-peptide, a protease domain, and frequently one or more additional domains [1, 4]. In prokaryotes, subtilases are generally secreted outside the cell during nutrition and play a role in host invasion. Subtilases lacking a signal peptide should remain inside the cell and most likely play a role in intracellular maturation of other proteins and peptides [4].

The first subtilase identified in eukaryotes was kexin [5]. Since then, nine subtilases have been discovered in mammals, among which seven are related to kexin and the remaining two (S1P and PCSK9) belong to the proteinase K and pyrolysin subfamilies of subtilases, respectively [6]. They are involved in the maturation of growth factors, neuropeptides, peptide hormones, receptor proteins, enzymes and viral surface glycoproteins in animals [7, 8]. The first subtilase cloned from higher plants was cucumisin, an extracellular protease highly abundant in melon fruit [9]. After that, other subtilase cDNAs have been cloned from *Alnus glutinosa*, *Arabidopsis thaliana* and *Lilium multiflorum* [10, 11]. The most striking characteristic of plant subtilases is the presence of long insertions of up to 169 amino acids in the central region of the catalytic domain, resulting in a shift of the Ser of the catalytic triad towards the C-terminus [1]. Moreover, the completion of genome projects for some model species revealed that large subtilase gene families exist throughout the plant kingdom, ranging from 23 genes in the moss *Physcomitrella patens*, 56 genes in *Arabidopsis* [12, 13], and 63 genes in rice [14] to 90 members in *Populus trichocarpa* [15]. Clearly, plants possess many more of these subtilases than animals, suggesting important roles of subtilases in plant biology. Plant subtilases share many properties with their bacterial and mamalian homologs, but have some unique biochemical and structural features (e.g. Ca^2+^ independence and the inserted PA_subtilisin_like domain) that distinguish them from those in other organisms [15–18]. Expansion of the subtilase family in plants is also accompanied by functional diversification. It seems that most plant subtilases have gained some plant-specific functions during their evolution. For instance plant subtilases are involved in xylem differentiation [19], fruit ripening [20], seed development [21, 22], formation of lateral roots [23] and pathogen interactions [24–26]. In addition, this gene family is also related the processing of peptide growth factors [27] and programmed cell death in plants [28, 29].

Structural features and expression profiles of some subtilase homologs have been partially described in *Arabidopsis* [12–14] and rice [14]. However, there is much less information about this gene family in *Vitis vinifera*. In the present study, we performed a genome-wide identification of the subtilase gene family in *V. vinifera*. Detailed analyses, including the molecular phylogeny, structural organization, functional divergence, adaptive evolution and expression profiling, were performed. Such an in-depth investigation is expected to provide insights into the underlying evolutionary mechanisms of the subtilase gene family in *V. vinifera*.

## Results and discussion

### Identification and characterization of the subtilase gene family in *V. vinifera*

Subtilases possess a conserved protease-associated (PA) subtilisin-like domain (PA_subtilisin_like domain). Based on this, we used the amino acid sequence of the PA_subtilisin_like domain (cd02120) as a query to search for homologs encoded by the grape genome. Subsequently, all identified candidate subtilase sequences were analyzed to determine whether they contain PA_subtilisin_like domains using the Conserved Domain Database (CDD) [30]. As a result, we identified 80 subtilase proteins in *V. vinifera* (Additional file 1: Table S1). This number is higher than those reported in other plant species, such as 56 in *Arabidopsis thaliana*, 63 in rice, and 15 in tomato [31]. These data likely suggest significant physiological functions of the subtilase gene family in *V. vinifera*. The numbers of subtilase genes in all plant species analyzed to date have been higher than in humans, suggesting their potentially more diverse function or different evolutionary mechanisms in plants.

Predictions of the subcellular localization of a gene product can provide additional information for its functional involvement. In this study, TargetP and PredoTar ( http://urgi.versailles.inra.fr/predotar/predotar.html) were used for primary structural analyses of grape subtilases [32]. The results indicated that most of the 80 grape subtilases possess signal sequences for targeting to the secretory pathway. In mammalian cells, most subtilases, such as proprotein convertases family (PCs), act as secretory enzymes and are targeted to the endoplasmic reticulum (ER) by virtue of their N-terminal signal peptides [1]. This result indicated a common feature among plant species, such as grape and mammalian cells. However, we found that 32 members of the subtilase genes in *V. vinifera* do not contain any known protein-targeting motif. It is predicted that two members (LOC100250428 and LOC100265894) are targeted to chloroplasts and one (LOC100255614) to mitochondria (Additional file 1: Table S1), suggesting potential chloroplast and mitochondrial functions. Different subcellular localizations of plant subtilases have been found to correlate with their different physiological functions. For example, the subtilase-like serine protease SDD1 in *Arabidopsis* is located at the cell plasma membrane, where it mediates cell-to-cell signaling and controls stomatal distribution and density during leaf development [33]. Another subtilisin-like protease, is stimulated in the presence of calcium ion [34]. ALE1 encodes a subtilisin-like serine protease, which is localized in the endosperm cells surrounding the embryo, and is required for epidermal surface formation in *Arabidopsis* embryos and juvenile plants [35]. In some cases, subtilases that share high sequence identities may have differential functions and are localized in specific tissues during different developmental stages. For example, tomato subtilase-like protease genes *P79A*, *P69B*, *P79C* and *P69D* share 79-88% identities; however, they exhibit different developmental and tissue-specific expression profiles, suggesting plant subtilases evolved various strategies to control their activities [36].

### Phylogenetic analyses of grape subtilases

**Figure 1:**
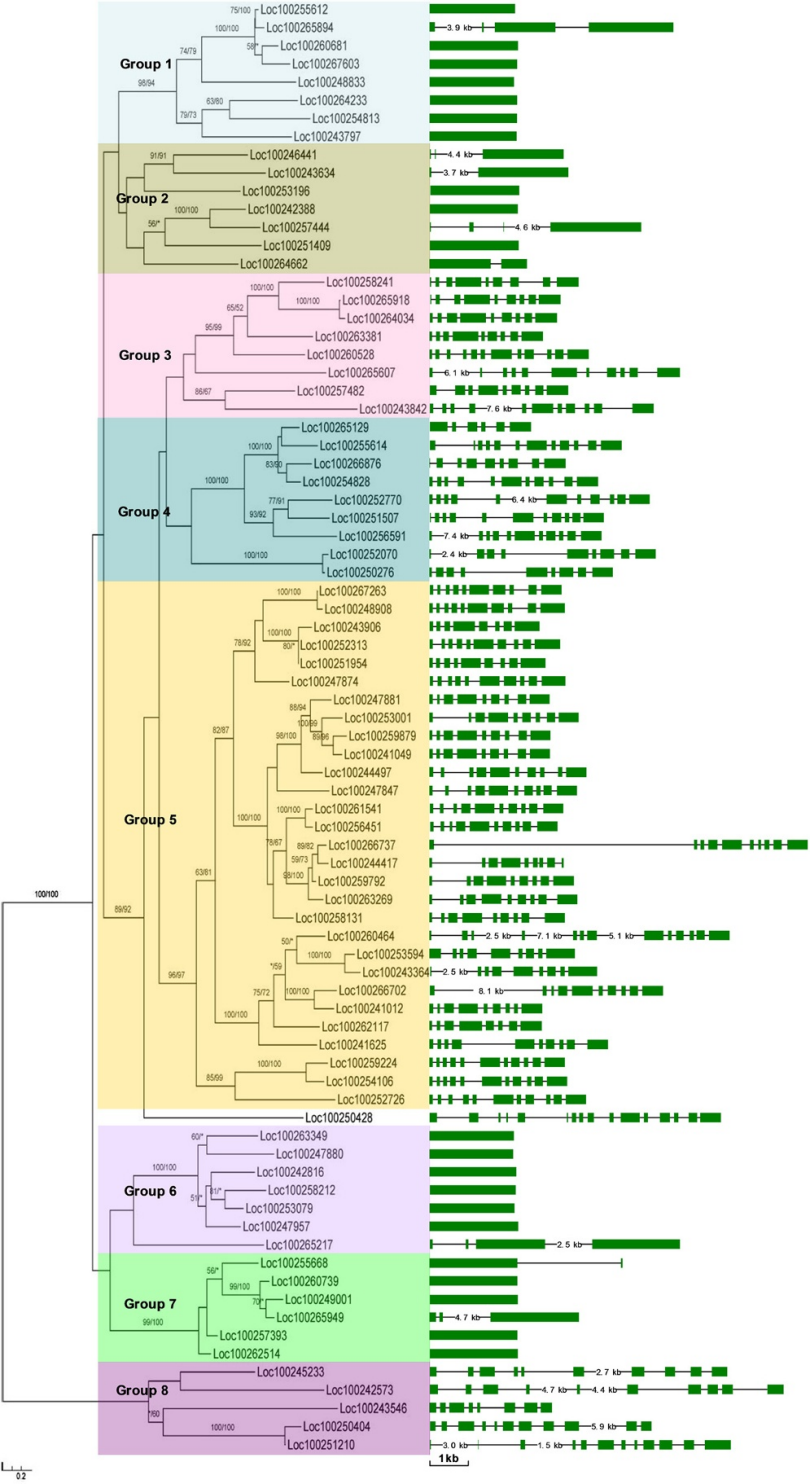
**Phylogenetic relationships and gene structures of grape subtilase genes.** The numbers above branches show bootstrap values from maximum likelihood (PhyML) and distance analyses (PHYLIP), respectively. The model used for ML analysis was LG + G, which was selected by ModelGenerator (AIC1). Eight major groups, designated 1 to 8, are marked with different colored backgrounds. The exon/intron structures of the subtilase genes are shown in the right panel. Green boxes represent exons and black lines represent introns.

**Figure 2:**
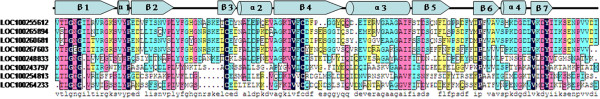
**Multiple sequence alignment of the PA-subtilisin-like domain of grape subtilase group 1.** The multiple alignment results clearly show the highly conserved PA-subtilisin-like domains among group 1 subtilase genes. The secondary structure elements of this domain are shown above the alignment. Cylindrical tubes represent α-helices and block arrows represent β-sheets.

Phylogenetic analyses of the 80 grape subtilases were performed, based on maximum likelihood and distance methods. The consensus phylogeny obtained from these analyses is shown in Figure 1. Based on their phylogenetic relationships, we divided the grape subtilase family into eight groups, designated group 1 to group 8 (Figure 1). The relationship of *LOC100250428* with the other subtilase genes, however, could not be confidently determined. Therefore, it was not classified into any group in this study. Another line of evidence, such as the gene organization as described below, supported the group classification of our analyses. Group 5, which contains 29 members, constitutes the largest clade in the subtilase phylogeny. Evolutionary relationships between the different groups of subtilase proteins could not be inferred. By contrast, the highly conserved amino acid sequence and gene organization suggested strong relationships between subfamily members within each group (see Figure 2). Our phylogenetic analyses also showed that several pairs of subtilase proteins are putative paralogs (Figure 1). These paralogous subtilase members are closely related and have a very similar structure (Figure 1), indicating that they evolved from relatively recent gene duplications. Similar to our analyses, a previous phylogenetic study of 56 *Arabidopsis* subtilases identified six distinct subfamilies (AtSBT1–AtSBT6), with five being similar to the pyrolysins and one more closely related to animal kexins [13]. AtSBT6 is the smallest of these subfamilies, and its two members appear to be more closely related to mammalian homologs than to other subtilases in *Arabidopsis* [13]. Recent findings show that AtSBT6.1 and 6.2 are in fact orthologs of the two mammalian pyrolysins and are involved in conserved processes across kingdoms [15]. Similarly, 15 subtilases of five distinct subfamilies were identified in the tomato (*Lycopersicon esculentum* Mill.) genome [37]; only one gene was found in the LeSBT1, LeSBT2 and tmp subfamilies; however, multiple members were identified in the LeSBT3/4 and P79 subfamilies. Within the LeSBT3/4 subfamily, LeSBT4A-E seemed to be more closely related to each other, indicating past gene duplication events in their evolution.

We also estimated the evolutionary dates of the segmental duplication events, using *K*_*s*_ as the proxy for time (Table 1, Additional file 1: Table S1). Seven of the 14 pairs (*LOC100244417*/*LOC100259792*, *LOC100256451*/*LOC100261541*, *LOC100241049*/*LOC100259879*, *LOC100241012*/*LOC100266702*, *LOC100243364*/*LOC100253594*, *LOC100260739*/*LOC100265949* and *LOC100250404*/*LOC100251210*) in grape have exceptionally consistent *K*_*s*_ values (from 0.08155 to 0.13598), suggesting that the duplication events occurred within the last 6.27 to 10.46 million years. Interestingly, two of the subtilase gene duplications (*LOC100251954*/*LOC100252313*, *LOC100264034*/*LOC100265918*) were estimated to have occurred more recently (only about 0.06 to 2.69 Ma). As shown in Additional file 1: Table S1, the duplication on one pair (*LOC100251507*/*LOC100252770*) was more ancient (about 90.33 Ma). This might reflect the macro-scale duplications and rearrangements between chromosome 12 and 19 (described below).

**Table 1:** **Inference of duplication time in paralogous pairs**

Paralogous pairs	***K***_***a***_	***K***_***s***_	Data (million years ago)
*LOC100244417/LOC100259792*	0.03677	0.10282	7.91
*LOC100256451/LOC100261541*	0.04231	0.10640	8.18
*LOC100241049/LOC100259879*	0.06607	0.10800	8.31
*LOC100251954/LOC100252313*	0.00208	0.00075	0.06
*LOC100241012/LOC100266702*	0.09410	0.10719	8.24
*LOC100243364/LOC100253594*	0.07714	0.12689	9.76
*LOC100254106/LOC100259224*	0.08763	0.18683	14.37
*LOC100250276/LOC100252070*	0.02462	0.06047	4.65
*LOC100251507/LOC100252770*	0.17495	1.17433	90.33
*LOC100264034/LOC100265918*	0.01839	0.03509	2.69
*LOC100255612/LOC100265894*	0.06835	0.27370	21.05
*LOC100260739/LOC100265949*	0.05576	0.08155	6.27
*LOC100257393/LOC100262514*	0.10259	0.34086	26.22
*LOC100250404/LOC100251210*	0.09847	0.13598	10.46

### Conserved and diverged domains, motifs and gene structures

The modular structure of subtilase proteins has been studied thoroughly in *Arabidopsis* [13]. This detailed information allowed us to analyze comparable domains for the 80 subtilases identified from the grape genome (Additional file 2: Table S2). We used CDD to identify major domains of subtilases in grape. Our results showed that four conserved domains (inhibitor_I9, peptidases_S8_3, PA_subtilisin_like, and peptidases_S8_S53) are present in the majority of grape subtilases (Additional file 2: Table S2). Compared with their mammalian homologs, plant subtilases share an insertion of 120–160 amino acids, within the catalytic domain (PA domain). The PA domain was originally identified as a region of homology between human transferrin and plant vacuolar sorting receptors. It is associated with different families of peptidases and has been implicated in protein–protein interactions and substrate recognition [15]. For most plant subtilases, such as tomato SlSBT3, their activation is stimulated by PA-domain-mediated homo-dimerization. In our analysis, most grape subtilases contained the PA domain, suggesting the potential function of the PA domain in grape subtilase protein dimerization [31], and that the ability to form homodimers through the PA domain is likely a common feature of plant subtilases. The PA domain is also important for determining optimum substrate length in soybean [38], suggesting a possible role of the PA domain in grape subtilases in substrate selection. Here, we also identified a novel domain, inhibitor_I9. This domain (sometimes referred to as an activation peptide) is responsible for modulating the folding and activity of the peptidase pro-enzyme. In many cases, it is synthesized as part of a large precursor protein as an N-terminal domain associated with an inactive peptidase. This domain prevents access of the substrate to the active site. Once the N-terminal inhibitory domain is removed, either by interaction with a secondary peptidase or by autocatalytic cleavage, the activity of subtilase is stimulated [39]. It seems that autocatalytic cleavage of the inhibitor-I9 domain contributes to the precise regulation of grape subtilase enzymes’ activities. A similar regulatory mechanism is reported in other plants. For example, the tomato (*Solanum lycopersicum*) SISBT3 possesses a potentially auto-inhibitory beta-hairpin domain that may obstruct the active site of the monomeric enzyme. Upon homo-dimerization mediated by the PA domain, this hairpin is immobilized by binding to the PA domain and its auto-inhibitory activity is relieved, stimulating the subtilase activity [40]. Peptidases_S8_3 and peptidases_S8_S53 domains might play a role in digesting the specific substrates for grape subtilases. However, the following exceptions were observed: in addition to the four conserved domains, LOC100253594, LOC100265894, LOC100265217, LOC100242573, LOC100245233, LOC100250404 and LOC100251210 also contain other domains. For example, the DUF1034 domain exists in LOC100245233 and LOC100251210. This domain functions in sugar hydrolysis in other organisms such as fungi [41]. In addition, the co-occurrence of a proteinase K domain and a P450 domain was reported in *Magnaporthe grisea* [41]. We also found that two subtilases (LOC100265129 and LOC100266876) do not contain the inhibitor_I9 domain. Three copies of the peptidases_S8_S53 domains were present in LOC100250428 (Additional file 2: Table S2), suggesting possible domain duplication events during this gene’s evolution.

**Figure 3:**
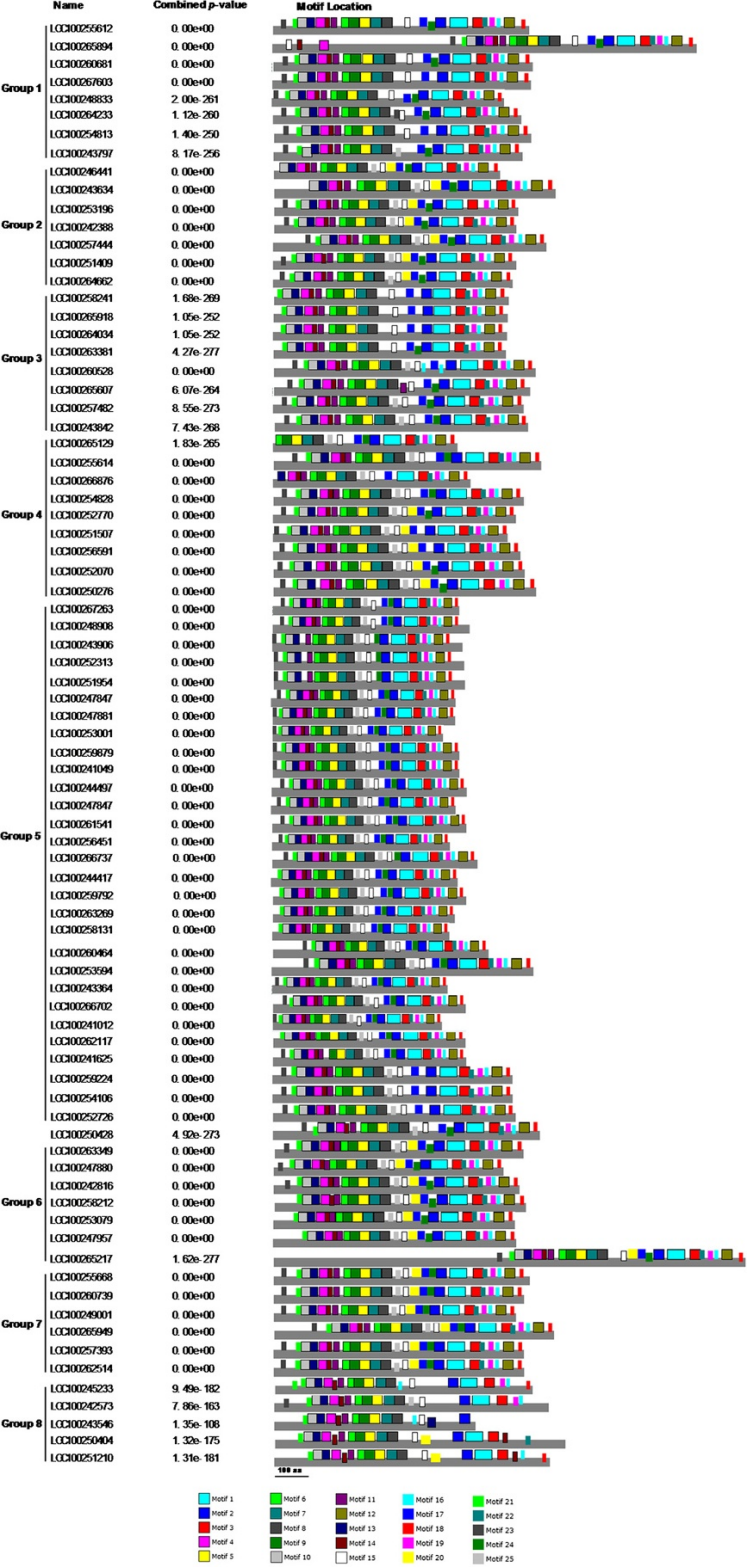
**Distribution of conserved motifs in the subtilase family members.** All motifs were identified by MEME using the complete amino acid sequence of the 80 grape subtilases documented in Figure 1. The names of all members among the defined gene clusters and combined *P*-values are shown on the left side of the figure; motif sizes are indicated at the bottom of the figure. Different motifs are indicated by different colors and are numbered 1–25. The same number in different proteins refers to the same motif. For details of the motifs refer to Table S3 (See Additional file 3).

CDD analyses were used to identify structurally conserved domains in subtilases. Small motifs and more divergent patterns cannot be recognized through CDD analyses; therefore, we also used MEME ( http://meme.sdsc.edu) [42] to study the diversification of grape subtilases. As a result, 25 distinct motifs were identified (Figure 3) and their details are presented in Additional file 3: Table S3. As mentioned above, phylogenetic analyses broadly divided grape subtilase genes into eight major groups. Noticeably, most of the closely related members in each of these groups have common motif compositions, suggesting functional similarities between the subtilase proteins within the same group. We also found that some motifs (motifs 12, 17 and 24) are absent from members of group 8, possibly leading to some functional differentiation. Generally, conserved motifs in subtilase proteins from the same group are consistent with the results of the phylogenetic analyses.

Gene structural diversification may play an important role in the evolution of multigene families [43, 44]. To gain further insights into the structural diversity of subtilases, we compared the exon-intron organization of the coding sequences of individual subtilase genes in grape. Detailed illustrations of the exon-intron structures are shown in Figure 1. In general, most closely related members in the same group shared a similar exon-intron structure. Interestingly, we also found that the number of introns varies considerably between different groups of grape subtilases, and most members of groups 1, 2, 6 and 7 do not contain introns. This can be explained by differences in the rates of intron gain and loss [45]. Similar to grape subtilases, papaya subtilases also contain introns [46]; however, intronless subtilase genes have been reported in *Arabidopsis* and tomato [31]. It has been suggested that introns not only increase the fitness of an organism by increasing intragenic recombination [45], but also are related to the evolutional rate of genes. For instance, some genes that rarely contain introns (F-box gene family, pentatricopeptide repeat containing gene family, DEAD box RNA helicases, early auxin-responsive SAUR) often experienced positive selection in their evolution [47–49]. Introns are unequally distributed in some gene families [50, 51] because of the ongoing intron gain and loss. Whether the large number of intron losses in groups 1, 2, 6 and 7 of grape subtilases have similar effects to those described above remains to be further experimentally examined.

### Chromosomal distribution and gene duplications of the grape subtilase genes

**Figure 4:**
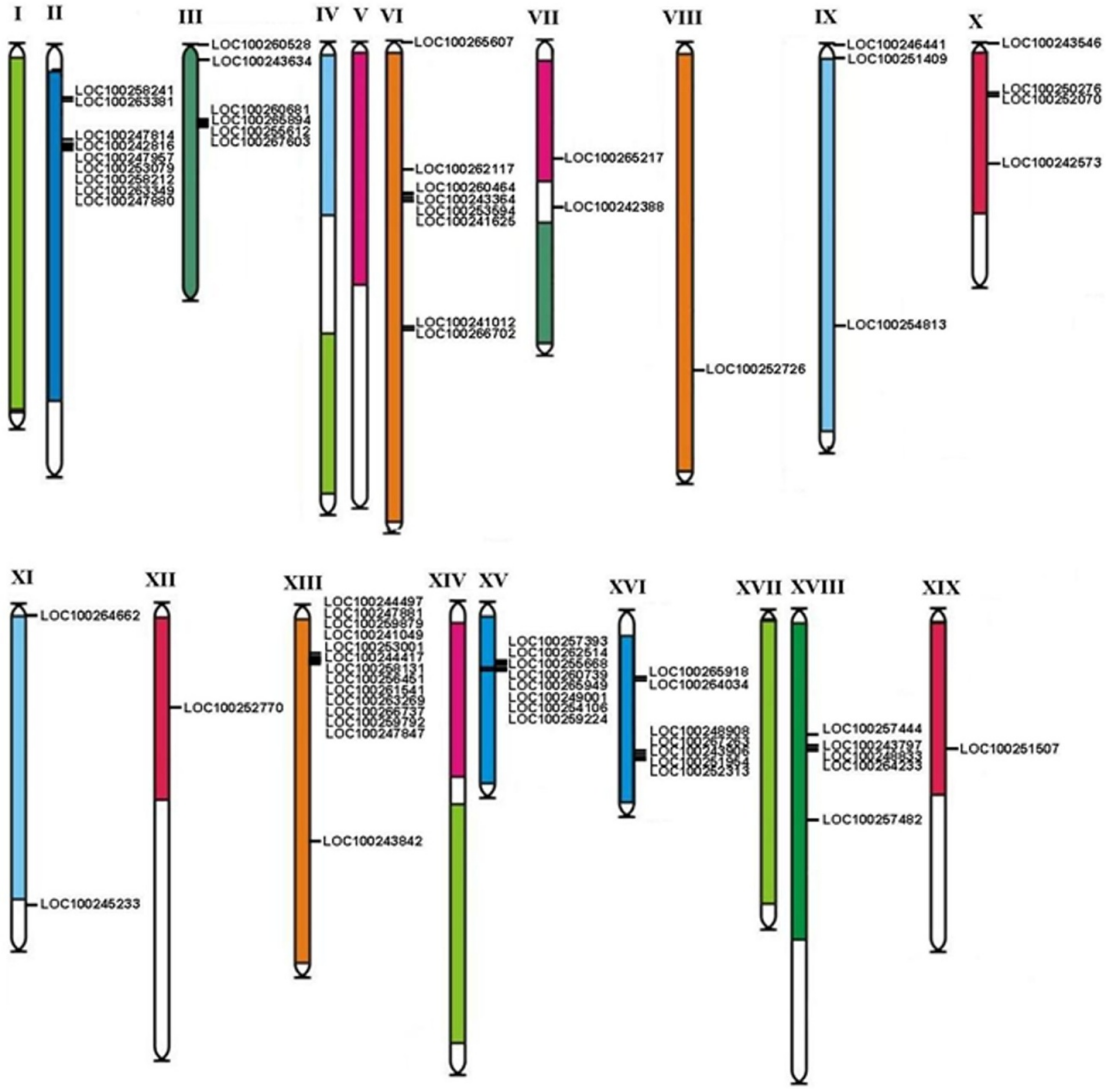
**Chromosomal locations of the grape subtilase genes.** The 71 subtilase genes mapping to 14 of the 19 grape chromosomes are shown. Paralogous regions in the putative ancestral constituents of the grape genome are depicted using the colors according to Jaillon *et al*. (2007) [48] and Licausi *et al*. (2010) [64].

**Figure 5:**
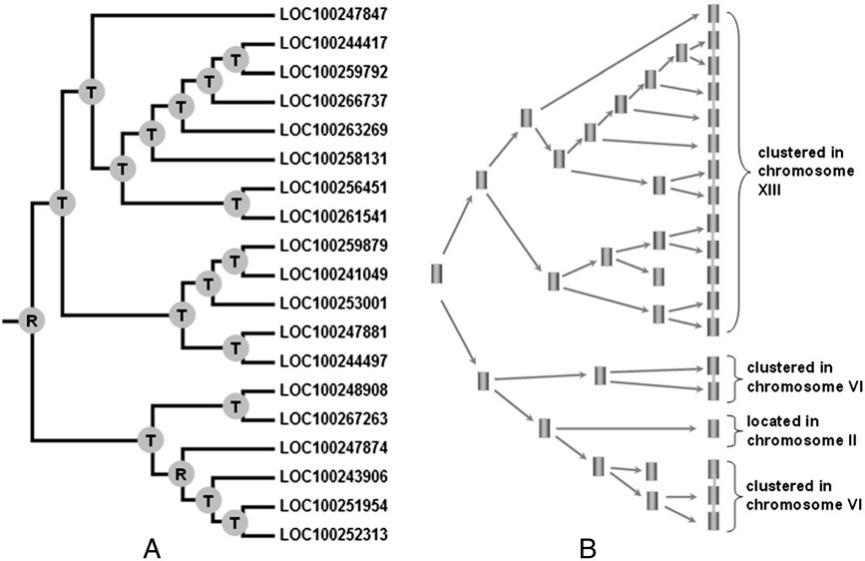
**Evolution of the one subgroup of grape subtilase genes.** **A**. Phylogenetic relationships. **B**. Hypothetical origins of 19 grape subtilase genes by tandem duplication and retroposition. The letters R and T on the nodes of the phylogenetic tree indicate the positions where retroposition and tandem duplication have occurred, respectively.

We further analyzed gene duplication events to understand the potential genetic mechanisms in the evolution of the grape subtilase gene family. First, we compared the locations of subtilase genes in duplicated chromosomal blocks that were previously identified in grape [52]. The distributions of subtilase genes relative to the corresponding duplicate genomic blocks are shown in Figure 4. Nine grape subtilase genes are located on unassembled genomic sequence scaffolds and thus could not be mapped to any particular chromosome. The other subtilase genes are distributed unevenly among 14 of the 19 grape chromosomes. Among the identified duplication events, only two subtilase genes (*LOC100251507* on XIX chromosome and *LOC100252770* on XII chromosome) are retained duplicates that are located in both duplicated chromosomal regions, whereas all others lack corresponding duplicates. From Figure 4, we also found that eight subtilase genes (*LOC100260528*, *LOC100265607*, *LOC100242388*, *LOC100246441*, *LOC100251409*, *LOC100243546*, *LOC100264662* and *LOC100245233*) are located outside of any duplicated blocks. This result suggests that segmental duplication is not the major factor that led to the expansion of the subtilase gene family in grape. It may be that dynamic changes occurred following segmental duplication, leading to the loss of many of the genes. Interestingly, we found that most subtilase genes are located in tandem clusters on the chromosomes. The largest subtilase gene cluster is located on chromosome 13 and contains 13 tandemly arrayed members, i.e. *LOC100247847*, *LOC100244417*, *LOC100259792*, *LOC100266737*, *LOC100263269*, *LOC100258131*, *LOC100256451*, *LOC100261541*, *LOC100259879*, *LOC100244049*, *LOC100253001*, *LOC100247881* and *LOC100244497* (Figures 4 and 5). Phylogenetically, these 13 genes form a single clade, suggesting that they may result from recent tandem duplications. However, we also found that five members (*LOC100248908*, *LOC10026726*3, *LOC1002439*06, *LOC100251954* and *LOC100252313*) on chromosome 6 and one member (*LOC100247874*) on chromosome 2 may also be derived from another duplication event of the 13-clustered members on chromosome 13. Further analyses indicate that most of these subtilases share relatively high similarities. We hypothesized that they might have resulted from more ancient tandem duplication or retroposition events (Figure 5). However, simultaneous expansions in the S8 and S53 families of subtilases in a single fungal species are rare [41]. In the *Arabidopsis* genome, 54 % of AtSBT genes also show tandem duplications of 2–5 genes. These arrangements suggest that local duplication events have also played an important role in the AtSBT family expansion. Furthermore, several highly similar sequences are found on different chromosomes. Similar situations indicative of a complex evolutionary history have been observed in other *Arabidopsis* gene families [13]. The MtSBT1.1 of *Medicago truncatula* show 90 % similarity to MtSB1.1 at the protein level, suggesting an ancestral duplication event [22]. Extracellular proteolytic activities of subtilase proteins are associated with virulence in pathogenic *Rhizopus oryzae*, and the whole genome duplication of subtilases in *R. oryzae* might have contributed to its virulence [53].

### Analysis of functional divergence

Could amino acid substitutions in subtilases have caused adaptive functional diversification? To answer this question, we estimated Type-I functional divergence between gene clusters of the grape subtilase family by posterior analysis using the program DIVERGE [54]. We compared 28 pairs of paralogous genes and estimated the rate of amino acid evolution at each sequence position. The results indicated that the coefficients of all functional divergence (θ) values between these groups were less than 1 (Table 2), suggesting site-specific selective constraints on most members of the grape subtilase family. Moreover, we also predicted some critical amino acid residues responsible for the functional divergence based on site-specific profiles, in combination with suitable cut-off values derived from the posterior probability of each comparison. These critical amino acid residues might have contributed to the functional divergence of grape subtilases. Similar to our analyses, Siezen et al. also found several essential and conserved amino acid residues, such as D32, H64 and G219, by comparing the PA domain in over 200 subtilases [55]. Our results also showed distinct differences in the number and distribution of predicted sites for functional divergence within each pair. For example, using a cut-off value of 0.5, only one critical amino acid site was predicted for the sequences in the group 3/8 pairs, while approximately 28 and 17 were predicted for group 3/6 and 3/7 pairs, respectively. As shown in Table 2, group 3/4 had the minimal theta value (θ) (0.0872), indicating the lowest evolutionary rate or site-specific selective relaxation between them. By contrast, the theta value in group 1/7 was the highest (0.5504), suggesting the largest divergence between them. Clearly, different evolutionary rates at specific sites within each pair could promote functional divergence among different groups during the long period of evolution. Our results showed that the different evolutionary rates at some important amino acid residues contributed to the evolution of grape subtilases, which might have acquired some group-specific functions.

**Table 2:** **Estimated functional divergence among grape subtilase paralogs**

Comparison	θ ^1^	SE ^2^	LRT ^3^	N(0.5) ^4^	N(0.7) ^4^
Group 1/Group 2	0.2792	0.088671	9.914529	17	10
Group 1/Group 3	0.28	0.087154	10.32146	20	6
Group 1/Group 4	0.2912	0.06808	18.29547	16	7
Group 1/Group 5	0.3632	0.050875	50.96668	28	21
Group 1/Group 6	0.21567	0.067419	10.23328	12	3
Group 1/Group 7	0.5504	0.137436	16.03827	102	14
Group 1/Group 8	0.2728	0.10368	6.92308	15	1
Group 2/Group 3	0.1944	0.066691	8.496944	7	2
Group 2/Group 4	0.2512	0.082217	9.335067	15	1
Group 2/Group 5	0.276	0.060202	21.01797	19	7
Group 2/Group 6	0.1576	0.049559	10.11273	9	3
Group 2/Group 7	0.4504	0.118338	14.48608	55	11
Group 2/Group 8	0.3208	0.089774	12.76937	55	5
Group 3/Group 4	0.0872	0.080381	1.176859	1	0
Group 3/Group 5	0.2528	0.04788	27.87704	14	9
Group 3/Group 6	0.3448	0.066534	26.85637	28	11
Group 3/Group 7	0.324	0.12143	7.119362	17	1
Group 3/Group 8	0.1432	0.103001	1.932856	1	0
Group 4/Group 5	0.2176	0.044709	23.68793	16	3
Group 4/Group 6	0.196	0.057016	11.81738	10	3
Group 4/Group 7	0.244	0.122626	3.959267	6	1
Group 4/Group 8	0.384	0.100291	14.66027	33	4
Group 5/Group 6	0.2744	0.038394	51.07865	19	12
Group 5/Group 7	0.2984	0.070052	18.14476	19	6
Group 5/Group 8	0.4992	0.078199	40.75143	74	24
Group 6/Group 7	0.4488	0.102943	19.0071	51	16
Group 6/Group 8	0.300968	0.079995	14.15512	21	2
Group 7/Group 8	0.5048	0.140123	12.97829	86	16

^1^θ is the coefficient of functional divergence.

^2^SE: standard error.

^3^LRT is a likelihood ratio test.

^4^N(0.5) and N(0.7) indicate the numbers of divergent residues when the cut-off values were 0.5 and 0.7, respectively.

### Site-specific selective pressure analysis

To analyze positive or negative selection of specific amino acid sites within the full-length sequences of the subtilase proteins in the different groups, substitution rate ratios of nonsynonymous (*K*_*a*_) versus synonymous (*K*_*s*_) mutations (the *K*_*a*_*/K*_*s*_ ratio measures selection pressure on amino acid substitutions) were calculated using Datamonkey [56]. Our results showed that the *K*_*a*_*/K*_*s*_ ratios of the sequences between subtilase groups were significantly different (Table 3). In addition, all the estimated *K*_*a*_*/K*_*s*_ values were substantially less than 1, suggesting that the subtilase sequences within each group are under strong purifying selection pressure. We performed the tests using three methods [SLAC (single likelihood ancestor counting), REL (random-effects likelihood) and FEL (fixed-effects likelihood)] [57]. The SLAC software detected no positively selected codon sites within groups 1, 2, 3, 4, 7 and 8, but found three and one positively selected sites within groups 5 and 6, respectively. FEL and REL analyses identified more sites (Table 3). The PARRIS test [58] did not reveal strong evidence (*P* < 0.001) of positive selection in subtilase coding sequences (Table 4). Clearly, although most of the protein residues are subjected to constant purifying selection, some sites have also been influenced by positive selection. Positive selection is an important adaptive mechanism; therefore, the sites under positive selection pressure might have accelerated functional divergence of grape subtilases, thus allowing the grape to adapt to its environment. Our results are in agreement with the study by Subbian et al. on the selective effect of subtilisin E and its homologous ISP proteins. Although subtilisin E and ISP are highly conserved in sequence and structure, they can fold through significantly different pathways and kinetics, and the positive selective effect on their surface residues could affect their thermodynamic stability and choice of folding pathways [59].

**Table 3:** **Predicted numbers and locations of codons under positive selection within different subtilase groups**

Gene branches	***K***_***a***_/***K***_***s***_	Positive selection sites			Integrative selection analysis
		SLAC	FEL	REL	Total number
Group 1	0.287208	-	634,656,**668**,801,**819**,**1074**,**1201**,1365,	598,615,617,623,647,**668**,729,**819**,835,846,867,868,932,996,999,1000,1017,1019,1022,1025,1034,1044,1046,1047,**1074**,1098,1131,1139,1140, 1193,**1201**,1303,	36
Group 2	0.233506	-	133,169,418,479,750,825,	-	6
Group 3	0.324256	-	31,59,118,122,182,216,284,472,480, 505,543,650,	-	12
Group 4	0.286081	-	2,56,436,501,651,718,821,	4,5,8,9,11,12,13,14,17,19,50,55,346, 804,	21
Group 5	0.3746	**152,255,321,**	49,108,**152**,166,**255**,315,**321**,326,468,**482**,514,**537**,564,569,577,	**255,321**,444,**482,537**,500,	17
Group 6	0.319197	**826,**	477,**486**,684,692,762,**795**,**826**,**942**,973,1067,1088,1139,	**486**,708,**795,826,942**,1090,	14
Group 7	0.407509	-	189,226,256,275,358,362,387,425,599,627,659,	92,122,247,249,255,343,349,432,451,529,533,634,674,708,733,819,827,847,861,	30
Group 8	0.348438	-	70,229,334,560,	10,14,43,46,49,51,780,792,800,818,822,823,825,836,838,839,842,843,846,852,853,859,865,874,876,936,937,940,941,942,	34

Bold codon sites indicate codons that were identified with at least two methods.

**Table 4:** **Evidence for positive selection in subtilase coding sequences**

Gene branches	Null model log-likelihoods	Alternative mode log-likelihoods	LRT	P value	Evidence for positive selection
Group 1	-17525.3	-17525.3	0	1	No
Group 2	-16363	-16363	0	1	No
Group 3	-17062	-17062	0	1	No
Group 4	-13388.4	13391.7	-6.6	1	No
Group 5	-36304.9	-36304.9	0	1	No
Group 6	-16140.6	-16140.6	0	1	No
Group 7	-8592.04	-8592.03	0.02	0.99005	No
Group 8	-12027.4	-12025.8	-16.8	1	No

LRT: likelihood ratio test.

### Differential expression profiles of grape subtilase genes

**Figure 6:**
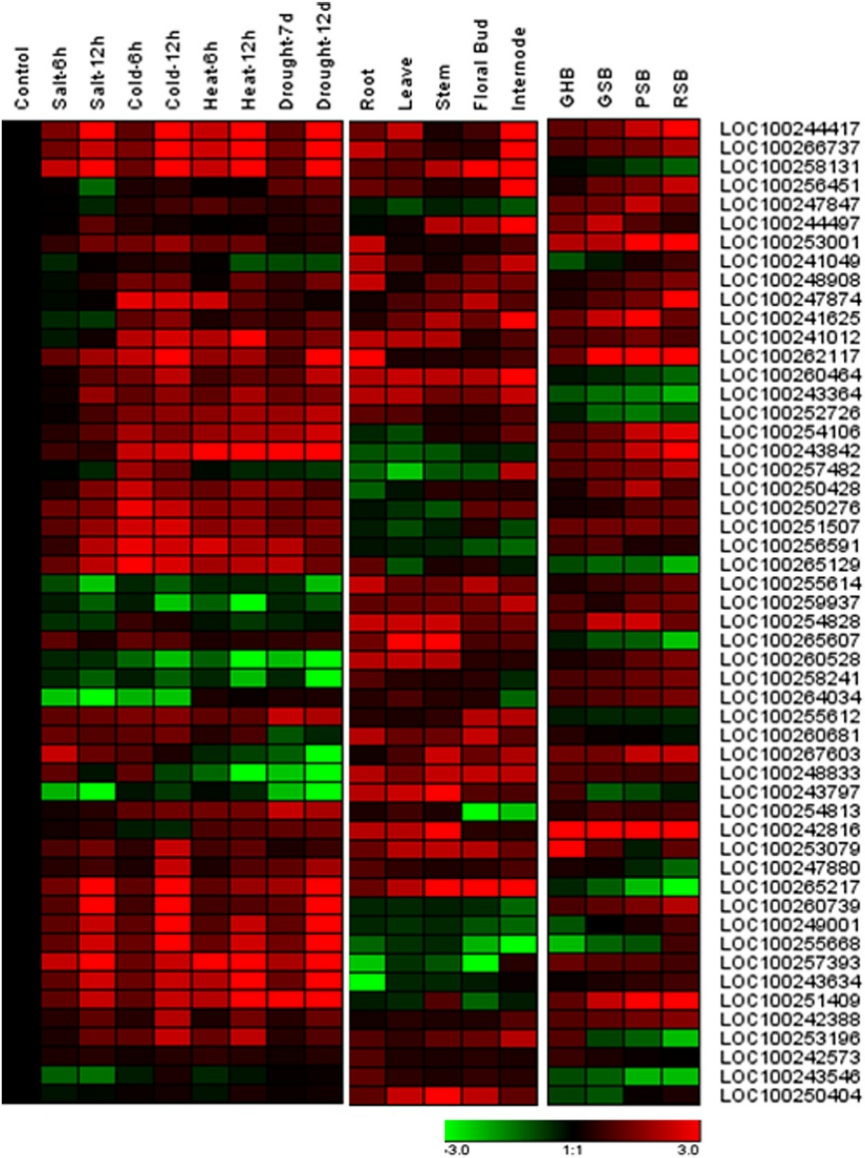
**Expression profiles of the grape subtilase gene family.** For saline stress treatments, 3-week grape seedlings were treated with 100 mM NaCl for 6 h or 12 h; for cold and heat stress treatments, the 3-week seedlings were treated with 4°C or 42°C for 6 h and 12 h; for drought treatments, the 3-week seedlings were dried for 7 days or 12 days. Expression profiles of subtilase genes family in different tissues (roots, leaves, stems, floral buds and internodes) and in 2-month-old grapes were used. GHB, green hard berry; GSB, green soft berry; PSB, pink soft berry; RSB, red soft berry.

Subtilases degrade substrates ranging from non-selective proteins to highly specific maturation of peptide hormones or protein precursors. Compared with animals, the subtilase family in plants has significantly expanded and has acquired some plant-specific functions. Plant subtilases are involved in stomata and seeds development, maintenance of shoot apical meristem and cell wall, processing of peptide growth factors, and response to abiotic environment [31]. Developmental or tissue-specific expressions of subtilases might represent various physiological functions [36]. Here, we first performed a comprehensive quantitative real-time-PCR (qRT-PCR) analysis of subtilases to investigate their expression patterns in different tissues. As shown in Figure 6, most subtilase genes show a constitutive distribution and slightly higher level of accumulation in grape leaves, root and shoot apices. Some subtilase genes, such as *LOC100260464*, *LOC100243364*, *LOC100248833*, *LOC100243797* and *LOC100265217*, are present at a ubiquitously high level in roots, leaves, stems, floral buds and internodes, indicating the role of subtilases in general tissue growth and development. In particular, we found that *LOC100258131* is highly similar to *Arabidopsis* subtilase SDD1 and a high transcript level in grape leaves, suggesting a similar function in leaf cell stomatal development as *Arabidopsis* SDD1 [33]. The *Arabidopsis* subtilase AIR3 is also highly expressed in lateral roots [60]. Similarly, *LOC100260681*, which is homologous to AIR3, also showed a high transcript level in grape roots. These gene transcription profiles suggested that some grape subtilases have similar expression patterns to their homologs in other plant species. *LOC100242816* showed the highest transcript level during all four phases of growth, despite its very low transcript level in floral buds and internode tissues.

We further selected four fruit growth phases to investigate these genes’ expressions during the fruit maturing process; these four phases were green hard berry, green soft berry, pink soft berry and red soft berry. As shown in Figure 6, different expression levels of subtilase genes were found in these four growth phases. Some genes, such as *LOC100251507* and *LOC100251409*, showed a higher transcript level in fruit, but lower in other tissues. Furthermore, *LOC100260464*, *LOC100243364*, *LOC100265217*, *LOC100243546* and *LOC100250404* showed lower transcript levels in fruit, but higher levels in leaves, roots and floral buds. The subtilase gene SBT1.1 is specifically expressed in the endosperm of *Medicago truncatula* and *Pisum sativum* seeds to control seed size [22]. In this study, we found that LOC 100253001, an ortholog of SBT1.1, was also transcribed at a high level during grape fruit development.

Environmental stress might regulate subtilase gene expression differentially [15]. Therefore, we tested the differences in expressions of grape subtilase mRNAs under various environmental stresses, including salt, cold, heat and drought. These environmental stresses are frequently confronted during grape growth. Several genes, such as *LOC100260739*, *LOC100249001*, *LOC100255668*, *LOC100257393*, *LOC100243634*, and *LOC100251409*, were obviously induced after different stress treatments. Other genes, including *LOC100255614*, *LOC100259937*, *LOC100254828*, *LOC100266528*, and *LOC100258241*, were obviously suppressed by these environmental stresses. Meanwhile, most of these genes demonstrated a comparable expression profile when subjected to the various environmental stresses, except *LOC100267603* and *LOC100248833*, which were expressed at a higher level after salt treatment, but a lower level after heat and drought stresses. Previous studies reported that PvSLP2 transcription was not induced by drought stress; however, PvSLP2 activity can be stimulated by drought stress, suggesting that plant subtilase activities may be regulated at the post-transcriptional level [61]. Thus, we could not exclude the possibility that some grape subtilases are involved in environmental stress responses at the post-transcriptional level, even though we did not detect their transcriptional differences.

## Conclusions

In summary, we identified and annotated 80 subtilases comprising eight subgroups in the *V. vinifera* genome. The analyses of gene structures, duplications, and selection provided valuable information on the evolution of grape subtilases. In particular, we found that tandem duplications have played an important role in the expansion of the subtilase gene family. Selection analysis revealed that purifying selection has been the main force during the evolution of the subtilase, while some of the critical sites have been subjected to positive selection. Moreover, analyses of their expression profiles provided functional information for members of the subtilase gene family in grape at different development stages. Further, investigations on the response patterns of the subtilase genes to salinity, cold, heat and drought conditions identified candidate stress-responsive genes in grape. Our results contribute valuable information for future functional investigations of this gene family.

## Methods

### Sequence retrieval and identification

To identify potential members of the subtilase gene family in grape, we performed multiple database searches. The amino acid sequence of the PA_subtilisin_like domain (cd02120) was retrieved and used as a query in BLAST searches against the grape genomes at the National Center for Biotechnology Information (NCBI, http://www.ncbi.nlm.nih.gov). TargetP and PredoTar ( http://urgi.versailles.inra.fr/predotar/predotar.html) were used for primary structure analyses of the grape subtilase members [32].

### Phylogenetic analyses of the grape subtilase gene family

Multiple sequence alignments of the full-length protein sequences were performed using MUSCLE 3.52, followed by manual comparisons and refinement [62]. Phylogenetic analyses of the subtilase protein family, based on amino acid sequences, were performed with a maximum likelihood method using PhyML 3.0 and by a distance method using PHYLIP [63]. ModelGenerator was used to select the optimal model of protein substitution and rate heterogeneity that best fitted the data set [64]. Bootstrap support values were estimated using 100 pseudo-replicates.

### Chromosomal location and gene structure of the subtilase genes

The chromosomal locations of the subtilase genes were determined using the grape genome browser ( http://www.genoscope.cns.fr/spip/Vitis-vinifera-e.html). Gene intron/exon structure information was collected from the genome annotations of grape from the NCBI and Phytozome ( http://www.phytozome.net) databases.

### Inference of duplication time

Pairwise alignment of nucleotide sequences of the subtilase paralogs was performed using MEGA 5 [65]. Alignments were performed using ClustalW (codons). The *K*_*a*_ and *K*_*s*_ values of the paralogous genes were estimated by the program K-Estimator 6.0 [61]. To better explain the patterns of macroevolution, estimates of the evolutionary rates were considered extremely useful. Assuming a molecular clock, the synonymous substitution rates (*K*_*s*_) of the paralogous genes are expected to be similar over time. Thus, *K*_*s*_ could be used as the proxy for time to estimate the dates of segmental duplication events. The *K*_*s*_ value was calculated for each of the gene pairs and then used to calculate the approximate date of the duplication event (T = *K*_*s*_/2λ), assuming clock-like rates (λ) of synonymous substitution of 6.5 × 10^- 9^ for grape [66].

### Conserved motifs analyses

The program MEME ( http://meme.sdsc.edu) was used to identify motifs in the candidate grape subtilase protein sequences [42]. MEME was run locally, with the following parameters: number of repetitions = any, maximum number of motifs = 25, and with optimum motif widths constrained to between six and 50 residues.

### Functional divergence analyses

To estimate the level of functional divergence and predict the amino acid residues responsible for functional differences in the subtilase subfamilies, coefficients of Type-I functional divergence were calculated using the method suggested by Gu [54]. The analyses were carried out with DINERGE (version 2.0). The method is based on maximum likelihood procedures to estimate significant changes in the site-specific shift of the evolutionary rate or the site-specific shift of amino acid properties after the emergence of two paralogous sequences. The advantage of this method is that it uses amino acid sequences and, therefore, is not sensitive to saturation of synonymous sites. Type-I functional divergence designates amino acid configurations that are highly conserved in gene 1 but highly variable in gene 2, or vice versa, implying that these residues have experienced altered functional constraints [54]. Coefficients of functional divergence that are significantly greater than 0 indicate site-specific altered selective constraints or radical shifts of amino acid physiochemical properties after gene duplication. Site-specific posterior analysis was used to predict amino acid residues that were crucial for functional divergence.

### Site-specific selection assessment and testing

In the study, SLAC, REL and FEL were employed to select individual codons using the default settings of the Datamonkey web interface [56, 57, 67]. SLAC fits a nucleotide substitution model to the data and calculates a global *Ka/Ks* ratio. Then, ancestral sequences at each codon are reconstructed using maximum likelihood. Finally, expected and observed numbers of synonymous and nonsynonymous substitutions are calculated to infer selection at each codon site. Significance was assessed using a *P* value derived from a two-tailed binomial distribution. SLAC calculates the expected and observed numbers of synonymous and nonsynonymous substitutions to infer selection. REL is an extension of the site-by-site positive selection analyses implemented in PAML [68]. Notably, it allows the synonymous and nonsynonymous substitution rates to vary among codon sites, and uses Bayes factors >50 to determine a site as selected [56, 67]. FEL directly estimates *Ka* and *Ks* based on a codon-substitution model; a likelihood ratio test is used to assess significance at a level of 0.1. Finally, we applied the "integrative selection analysis" to determine the total number of positively selected codons, which were detected by at least one of the three methods [56, 67]. PARRIS can allow tree topologies and branch lengths to change across detected recombination breakpoints [58]; therefore, we used it to test for the signatures of selection.

### RNA extraction and real-time qRT-PCR

Total RNA after different stress treatments or from different tissues was isolated using an RNeasy Kit (Qiagen) from plant samples that had been ground in liquid nitrogen and then converted into first-strand cDNA using SuperScriptII reverse transcriptase (Invitrogen) with an oligo(dT) primer. The cDNA templates were amplified using a CFX384 Real-time PCR detection system (Bio-Rad) with SYBR premix Ex Taq (Takara). The primer sequences are given in Additional file 4: Table S4. The thermal program was 5 min at 95°C, followed by 60 cycles of 10 s at 95°C, 10 s at 55°C and 10 s at 72°C. The specificity of the reactions was confirmed by the machine standard melt curve method. The grape tubulin gene was used as the reference gene. The quantified data were analyzed by hierarchical clustering using the cluster 3.0 and Treeview software ( http://bonsai.ims.u-tokyo.ac.jp/~mdehoon/software/cluster). The different colors correspond to the log-transformed values of protein change-fold ratios shown in the bar at the bottom of Figure 6.

## Electronic supplementary material

Additional file 1: Table S1: Targeting prediction of the 80 grape subtilases, using either TargetP V1.1 or PredoTar V1.03. cTP: chloroplast transit peptide; mTP: mitochondrial targeting peptide; SP: secretory pathway signal peptide; S: secretory pathway; M: mitochondria; C: chloroplast; ER: endoplasmic reticulum. (DOC 220 KB)

Additional file 2: Table S2: Functional domains of grape subtilases. The numbers in brackets indicate E-values. (DOC 176 KB)

Additional file 3: Table S3: Motif sequences identified by MEME tools. The numbers correspond to the motifs described in Figure 3. (DOC 46 KB)

Additional file 4: Table S4: Primers used for quantitative real-time qRT-PCR of *Vitis vinifera.* (DOC 110 KB)
