# Supplementary material for: Genome-wide and molecular evolution analysis of the subtilase gene family in Vitis vinifera
Source: BMC Genomics. 2014 Dec 16;15(1):1116. doi: 10.1186/1471-2164-15-1116 (PMC4378017; doi:10.1186/1471-2164-15-1116)
Supplement: Supplementary file 1 — Additional file 1: Table S1: Targeting prediction of the 80 grape subtilases, using either TargetP V1.1 or PredoTar V1.03. cTP: chloroplast transit peptide; mTP: mitochondrial targeting peptide; SP: secretory pathway signal peptide; S: secretory pathway; M: mitochondria; C: chloroplast; ER: endoplasmic reticulum. (DOC 220 KB) [file 12864_2014_6873_MOESM1_ESM.doc]

**Table S1**. Targeting prediction of the 80 grape subtilases using either TargetP V1.1 or PredoTar V1.03.

|  |  | **TargetP V1.1 Prediction Results** | | | | | **PredoTar V1.03 Prediction Results** | | | | |
| --- | --- | --- | --- | --- | --- | --- | --- | --- | --- | --- | --- |
| **Name** | **Length** | **cTP** | **mTP** | **SP** | **Other** | **Loc** | **Mitochondrial** | **Plastid** | **ER** | **Elsewhere** | **Prediction** |
| LOC100241012 | 689 | 0.211 | 0.095 | 0.032 | 0.712 | - | 0.01 | 0.01 | 0.04 | 0.94 | none |
| LOC100241049 | 712 | 0.152 | 0.202 | 0.087 | 0.587 | - | 0.01 | 0.00 | 0.01 | 0.98 | none |
| LOC100241625 | 737 | 0.002 | 0.041 | 0.890 | 0.205 | S | 0.02 | 0.00 | 0.95 | 0.05 | ER |
| LOC100242388 | 767 | 0.005 | 0.164 | 0.754 | 0.136 | S | 0.01 | 0.00 | 0.99 | 0.01 | ER |
| LOC100242573 | 842 | 0.004 | 0.013 | 0.987 | 0.244 | S | 0.01 | 0.01 | 0.99 | 0.01 | ER |
| LOC100242816 | 755 | 0.043 | 0.014 | 0.879 | 0.109 | S | 0.02 | 0.00 | 0.99 | 0.01 | ER |
| LOC100243364 | 713 | 0.055 | 0.090 | 0.096 | 0.843 | - | 0.01 | 0.02 | 0.04 | 0.94 | none |
| LOC100243546 | 602 | 0.003 | 0.096 | 0.962 | 0.148 | S | 0.01 | 0.00 | 0.99 | 0.01 | ER |
| LOC100243634 | 802 | 0.006 | 0.340 | 0.568 | 0.099 | S | 0.47 | 0.01 | 0.71 | 0.15 | ER |
| LOC100243797 | 763 | 0.001 | 0.178 | 0.896 | 0.043 | S | 0.01 | 0.00 | 0.99 | 0.01 | ER |
| LOC100243842 | 777 | 0.018 | 0.008 | 0.980 | 0.028 | S | 0.01 | 0.00 | 0.99 | 0.01 | ER |
| LOC100243906 | 700 | 0.146 | 0.179 | 0.086 | 0.608 | - | 0.01 | 0.00 | 0.02 | 0.98 | none |
| LOC100244417 | 698 | 0.165 | 0.186 | 0.082 | 0.386 | - | 0.01 | 0.18 | 0.00 | 0.81 | none |
| LOC100244497 | 742 | 0.014 | 0.045 | 0.966 | 0.016 | S | 0.04 | 0.00 | 0.99 | 0.01 | ER |
| LOC100245233 | 817 | 0.004 | 0.032 | 0.990 | 0.083 | S | 0.01 | 0.00 | 0.99 | 0.01 | ER |
| LOC100246441 | 718 | 0.108 | 0.449 | 0.015 | 0.565 | - | 0.01 | 0.00 | 0.00 | 0.99 | none |
| LOC100247847 | 731 | 0.014 | 0.055 | 0.817 | 0.150 | S | 0.01 | 0.00 | 0.97 | 0.03 | ER |
| LOC100247874 | 766 | 0.009 | 0.040 | 0.958 | 0.046 | S | 0.02 | 0.00 | 0.99 | 0.01 | ER |
| LOC100247880 | 728 | 0.164 | 0.038 | 0.052 | 0.941 | - | 0.01 | 0.00 | 0.01 | 0.98 | none |
| LOC100247881 | 696 | 0.049 | 0.072 | 0.298 | 0.850 | - | 0.01 | 0.00 | 0.00 | 0.99 | none |
| LOC100247957 | 772 | 0.003 | 0.055 | 0.990 | 0.033 | S | 0.01 | 0.00 | 0.96 | 0.04 | ER |
| LOC100248833 | 736 | 0.413 | 0.027 | 0.034 | 0.874 | - | 0.01 | 0.01 | 0.00 | 0.99 | none |
| LOC100248908 | 773 | 0.001 | 0.212 | 0.948 | 0.020 | S | 0.03 | 0.00 | 0.99 | 0.01 | ER |
| LOC100249001 | 768 | 0.011 | 0.049 | 0.983 | 0.057 | S | 0.02 | 0.00 | 0.99 | 0.01 | ER |
| LOC100250276 | 778 | 0.006 | 0.274 | 0.880 | 0.015 | S | 0.02 | 0.00 | 0.99 | 0.01 | ER |
| LOC100250404 | 890 | 0.008 | 0.031 | 0.969 | 0.106 | S | 0.01 | 0.02 | 0.90 | 0.09 | ER |
| LOC100250428 | 819 | 0.456 | 0.119 | 0.260 | 0.341 | C | 0.01 | 0.12 | 0.00 | 0.87 | none |
| LOC100251210 | 845 | 0.019 | 0.038 | 0.872 | 0.256 | S | 0.01 | 0.00 | 0.99 | 0.01 | ER |
| LOC100251409 | 771 | 0.087 | 0.003 | 0.905 | 0.071 | S | 0.01 | 0.04 | 0.99 | 0.01 | ER |
| LOC100251507 | 745 | 0.050 | 0.308 | 0.170 | 0.737 | - | 0.01 | 0.01 | 0.01 | 0.97 | none |
| LOC100251954 | 718 | 0.148 | 0.182 | 0.075 | 0.607 | - | 0.01 | 0.00 | 0.02 | 0.98 | none |
| LOC100252070 | 769 | 0.045 | 0.059 | 0.355 | 0.608 | - | 0.01 | 0.00 | 0.10 | 0.90 | none |
| LOC100252313 | 744 | 0.046 | 0.080 | 0.631 | 0.098 | S | 0.01 | 0.00 | 0.99 | 0.01 | ER |
| LOC100252726 | 744 | 0.012 | 0.038 | 0.895 | 0.187 | S | 0.01 | 0.00 | 0.99 | 0.01 | ER |
| LOC100252770 | 769 | 0.002 | 0.072 | 0.991 | 0.017 | S | 0.02 | 0.00 | 0.99 | 0.01 | ER |
| LOC100253001 | 696 | 0.074 | 0.110 | 0.145 | 0.868 | - | 0.01 | 0.00 | 0.00 | 0.99 | none |
| LOC100253079 | 740 | 0.126 | 0.038 | 0.798 | 0.185 | S | 0.01 | 0.01 | 0.99 | 0.01 | ER |
| LOC100253196 | 774 | 0.007 | 0.127 | 0.955 | 0.008 | S | 0.04 | 0.00 | 0.99 | 0.01 | ER |
| LOC100253594 | 802 | 0.018 | 0.027 | 0.922 | 0.175 | S | 0.01 | 0.00 | 0.13 | 0.86 | none |
| LOC100254106 | 737 | 0.007 | 0.024 | 0.983 | 0.075 | S | 0.01 | 0.01 | 0.83 | 0.17 | ER |
| LOC100254813 | 762 | 0.009 | 0.016 | 0.961 | 0.072 | S | 0.01 | 0.01 | 0.92 | 0.08 | ER |
| LOC100254828 | 767 | 0.003 | 0.046 | 0.979 | 0.058 | S | 0.01 | 0.00 | 0.95 | 0.05 | ER |
| LOC100255612 | 762 | 0.016 | 0.020 | 0.982 | 0.043 | S | 0.01 | 0.00 | 0.99 | 0.01 | ER |
| LOC100255614 | 793 | 0.070 | 0.264 | 0.173 | 0.192 | M | 0.06 | 0.01 | 0.50 | 0.47 | ER |
| LOC100255668 | 786 | 0.009 | 0.123 | 0.935 | 0.056 | S | 0.02 | 0.00 | 0.99 | 0.01 | ER |
| LOC100256451 | 727 | 0.009 | 0.028 | 0.920 | 0.245 | S | 0.02 | 0.01 | 0.99 | 0.01 | ER |
| LOC100256591 | 758 | 0.047 | 0.358 | 0.122 | 0.579 | - | 0.01 | 0.00 | 0.00 | 0.99 | none |
| LOC100257393 | 768 | 0.017 | 0.135 | 0.936 | 0.021 | S | 0.15 | 0.00 | 0.99 | 0.01 | ER |
| LOC100257444 | 837 | 0.041 | 0.095 | 0.829 | 0.068 | S | 0.01 | 0.00 | 0.99 | 0.01 | ER |
| LOC100257482 | 768 | 0.024 | 0.089 | 0.790 | 0.080 | S | 0.01 | 0.00 | 0.99 | 0.01 | ER |
| LOC100258131 | 697 | 0.100 | 0.161 | 0.086 | 0.777 | - | 0.01 | 0.03 | 0.00 | 0.97 | none |
| LOC100258212 | 744 | 0.019 | 0.008 | 0.979 | 0.168 | S | 0.01 | 0.02 | 0.99 | 0.01 | ER |
| LOC100258241 | 720 | 0.226 | 0.071 | 0.091 | 0.565 | - | 0.01 | 0.03 | 0.00 | 0.96 | none |
| LOC100259224 | 736 | 0.003 | 0.038 | 0.985 | 0.064 | S | 0.01 | 0.00 | 0.98 | 0.02 | ER |
| LOC100259792 | 701 | 0.108 | 0.250 | 0.056 | 0.423 | - | 0.01 | 0.39 | 0.00 | 0.60 | possibly plastid |
| LOC100259879 | 705 | 0.333 | 0.103 | 0.126 | 0.594 | - | 0.01 | 0.01 | 0.00 | 0.98 | none |
| LOC100260464 | 818 | 0.024 | 0.315 | 0.335 | 0.430 | - | 0.20 | 0.02 | 0.01 | 0.77 | none |
| LOC100260528 | 802 | 0.259 | 0.030 | 0.257 | 0.377 | - | 0.01 | 0.62 | 0.11 | 0.34 | plastid |
| LOC100260681 | 769 | 0.007 | 0.017 | 0.992 | 0.056 | S | 0.01 | 0.00 | 0.99 | 0.01 | ER |
| LOC100260739 | 767 | 0.006 | 0.063 | 0.980 | 0.089 | S | 0.04 | 0.00 | 0.99 | 0.01 | ER |
| LOC100261541 | 735 | 0.021 | 0.022 | 0.713 | 0.215 | S | 0.02 | 0.01 | 0.99 | 0.01 | ER |
| LOC100262117 | 703 | 0.223 | 0.179 | 0.084 | 0.457 | - | 0.01 | 0.00 | 0.00 | 0.99 | none |
| LOC100262514 | 767 | 0.021 | 0.110 | 0.909 | 0.080 | S | 0.03 | 0.00 | 0.99 | 0.01 | ER |
| LOC100263269 | 697 | 0.156 | 0.117 | 0.090 | 0.590 | - | 0.01 | 0.11 | 0.00 | 0.89 | none |
| LOC100263349 | 737 | 0.080 | 0.037 | 0.840 | 0.131 | S | 0.03 | 0.01 | 0.99 | 0.01 | ER |
| LOC100263381 | 737 | 0.095 | 0.089 | 0.072 | 0.928 | - | 0.01 | 0.00 | 0.00 | 0.99 | none |
| LOC100264034 | 742 | 0.082 | 0.103 | 0.042 | 0.921 | - | 0.01 | 0.00 | 0.02 | 0.97 | none |
| LOC100264233 | 762 | 0.006 | 0.019 | 0.969 | 0.063 | S | 0.02 | 0.00 | 0.99 | 0.01 | ER |
| LOC100264662 | 761 | 0.025 | 0.233 | 0.865 | 0.009 | S | 0.02 | 0.00 | 0.99 | 0.01 | ER |
| LOC100265129 | 565 | 0.052 | 0.505 | 0.023 | 0.736 | - | - | - | - | - | - |
| LOC100265217 | 1413 | 0.008 | 0.070 | 0.961 | 0.187 | S | 0.01 | 0.01 | 0.98 | 0.02 | ER |
| LOC100265607 | 788 | 0.052 | 0.255 | 0.177 | 0.885 | - | 0.01 | 0.00 | 0.07 | 0.93 | none |
| LOC100265894 | 1361 | 0.644 | 0.091 | 0.007 | 0.359 | C | 0.01 | 0.18 | 0.01 | 0.80 | none |
| LOC100265918 | 717 | 0.210 | 0.110 | 0.038 | 0.633 | - | 0.04 | 0.02 | 0.00 | 0.95 | none |
| LOC100265949 | 858 | 0.506 | 0.146 | 0.114 | 0.520 | - | 0.01 | 0.01 | 0.00 | 0.99 | none |
| LOC100266702 | 735 | 0.234 | 0.060 | 0.056 | 0.896 | - | 0.01 | 0.01 | 0.00 | 0.99 | none |
| LOC100266737 | 705 | 0.065 | 0.108 | 0.090 | 0.909 | - | 0.01 | 0.00 | 0.00 | 0.99 | none |
| LOC100266876 | 628 | 0.088 | 0.193 | 0.135 | 0.784 | - | 0.01 | 0.00 | 0.00 | 0.99 | none |
| LOC100267263 | 736 | 0.001 | 0.212 | 0.948 | 0.020 | S | 0.03 | 0.00 | 0.99 | 0.01 | ER |
| LOC100267603 | 765 | 0.004 | 0.033 | 0.990 | 0.037 | S | 0.01 | 0.00 | 0.99 | 0.01 | ER |

cTP: chloroplast transit peptide; mTP: mitochondrial targeting peptide; SP: secretory pathway signal peptide; S: secretory pathway; M: mitochondria; C: chloroplast; ER: endoplasmic reticulum.
