# Supplementary material for: Genome-wide and molecular evolution analysis of the subtilase gene family in Vitis vinifera
Source: BMC Genomics. 2014 Dec 16;15(1):1116. doi: 10.1186/1471-2164-15-1116 (PMC4378017; doi:10.1186/1471-2164-15-1116)
Supplement: Supplementary file 2 — Additional file 2: Table S2: Functional domains of grape subtilases. The numbers in brackets indicate E-values. (DOC 176 KB) [file 12864_2014_6873_MOESM2_ESM.doc]

**Table S2**. Functional domains of grape subtilases.

| **Gene name** | **Group** | **Inhibitor_I9** | **Peptidases_S8_3** | **PA_subtilisin_like** | **Peptidases_S8_S53** | **Others domain** |
| --- | --- | --- | --- | --- | --- | --- |
| LOC100255612 | 1 | 25-105 (7.70e-15) | 103-355 (5.09e-107) | 347-466 (2.23e-15) | 497-571 (1.25e-33) | |
| LOC100265894 | 1 | 625-704 (1.55e-13) | 702-952 (5.58e-107) | 944-1065 (2.53e-17) | 1096-1170 (5.13e-33) | **Limkani_b1_N_like** [115-261 (6.67e-57)]**;**  **LOTUS** [381-450 (1.09e-17)]**;**  **LOTUS** [525-587 (6.53e-14)] |
| LOC100260681 | 1 | 29-109 (3.93e-16) | 107-360 (3.27e-110) | 352-473 (2.18e-15) | 504-578 (4.10e-32) | |
| LOC100267603 | 1 | 29-105 (2.04e-14) | 109-349 (6.76e-110) | 350-470 (6.52e-14) | 496-577 (1.36e-31) | |
| LOC100248833 | 1 | 8-75 (5.78e-15) | 87-321 (3.77e-92) | 328-447 (3.32e-15) | 471-554 (8.95e-30) | |
| LOC100264233 | 1 | 27-116 (3.53e-14) | 113-344 (3.39e-74) | 352-465 (5.55e-13) | 496-570 (7.57e-31) | |
| LOC100254813 | 1 | 30-114 (3.75e-16) | 111-343 (9.03e-91) | 350-463 (5.13e-10) | 493-567 (1.22e-31) | |
| LOC100243797 | 1 | 33-120 (4.05e-16) | 117-348 (1.88e-107) | 355-471 (9.70e-10) | 500-576 (5.54e-29) | |
| LOC100246441 | 2 | 14-49 (2.74e-05) | 47-305 (4.03e-101) | 288-421 (3.35e-33) | 452-526 (5.21e-36) | |
| LOC100243634 | 2 | 59-132 (1.16e-14) | 130-373 (1.03e-90) | 380-506 (7.00e-35) | 537-611 (1.40e-36) | |
| LOC100253196 | 2 | 30-116 (1.95e-12) | 113-348 (4.61e-106) | 355-485 (1.81e-33) | 516-590 (1.22e-37) | |
| LOC100242388 | 2 | 34-109 (7.76e-15) | 106-339 (6.63e-106) | 347-473 (1.78e-31) | 504-578 (5.90e-35) | |
| LOC100257444 | 2 | 102-178 (8.89e-16) | 175-408 (5.49e-117) | 416-542 (5.61e-37) | 555-647 (4.53e-35) | |
| LOC100251409 | 2 | 27-106 (1.81e-16) | 103-339 (6.92e-108) | 346-469 (9.90e-31) | 473-575 (4.87e-34) | |
| LOC100264662 | 2 | 24-100 (1.07e-17) | 97-338 (7.32e-88) | 348-467 (1.79e-34) | 500-574 (5.83e-35) | |
| LOC100258241 | 3 | 7-57 (3.64e-13) | 54-300 (2.72e-103) | 308-432 (1.74e-23) | 459-532 (3.42e-33) | |
| LOC100265918 | 3 | 3-48 (2.84e-12) | 54-301 (2.33e-99) | 309-427 (2.66e-18) | 462-529 (9.64e-27) | |
| LOC100264034 | 3 | 1-72 (5.81e-15) | 94-317 (2.66e-93) | 325-456 (2.05e-16) | 487-554 (2.63e-27) | |
| LOC100263381 | 3 | 4-75 (6.36e-17) | 72-317 (2.91e-102) | 325-452 (3.19e-25) | 483-550 (1.55e-33) | |
| LOC100260528 | 3 | 58-138 (7.49e-20) | 137-375 (1.58e-76) | 382-510 (1.17e-22) | 542-619 (1.04e-31) | |
| LOC100265607 | 3 | 43-123 (3.77e-18) | 121-365 (2.89e-105) | 373-488 (3.72e-10) | 514-594 (3.85e-37) | |
| LOC100257482 | 3 | 32-106 (1.20e-18) | 103-340 (5.11e-92) | 347-478 (4.22e-23) | 509-582 (1.20e-33) | |
| LOC100243842 | 3 | 31-114 (3.16e-14) | 111-354 (7.10e-99) | 361-491 (5.13e-24) | 522-596 (8.62e-29) | |
| LOC100265129 | 4 | - | 6-139 (1.46e-66) | 146-280 (1.16e-19) | 310-384 (4.01e-31) | |
| LOC100255614 | 4 | 56-143 (1.52e-11) | 140-373 (3.68e-96) | 380-510 (2.49e-22) | 540-613 (3.65e-31) | |
| LOC100266876 | 4 | - | 19-250 (5.43e-86) | 257-380 (2.27e-25) | 402-439 (4.26e-17) | |
| LOC100254828 | 4 | 28-115 (2.54e-07) | 113-345 (4.04e-93) | 352-482 (1.37e-25) | 510-585 (2.88e-29) | |
| LOC100252770 | 4 | 29-114 (9.01e-17) | 112-350 (2.87e-101) | 357-488 (1.19e-22) | 519-592 (1.37e-32) | |
| LOC100251507 | 4 | 6-91 (1.94e-09) | 89-327 (1.79e-100) | 334-465 (1.48e-30) | 496-569 (1.62e-32) | |
| LOC100256591 | 4 | 22-108 (5.41e-09) | 106-339 (1.17e-110) | 346-476 (8.07e-30) | 501-581 (2.90e-31) | |
| LOC100252070 | 4 | 15-90 (4.79e-17) | 94-349 (2.90e-109) | 357-488 (1.30e-37) | 519-592 (2.90e-32) | |
| LOC100250276 | 4 | 22-98 (1.48e-17) | 102-357 (3.00e-108) | 365-496 (4.32e-36) | 527-600 (4.95e-32) | |
| LOC100267263 | 5 | 31-110 (8.50e-17) | 107-382 (2.32e-88) | 337-459 (4.73e-15) | 489-563 (2.11e-34) | |
| LOC100248908 | 5 | 31-110 (1.12e-16) | 107-382 (3.80e-88) | 337-459 (3.46e-15) | 489-563 (1.48e-33) | |
| LOC100243906 | 5 | 2-81 (1.54e-16) | 78-282 (6.05e-67) | 290-353 (1.04e-06) | 410-484 (5.95e-35) | |
| LOC100252313 | 5 | 28-107 (1.94e-17) | 104-326 (5.77e-90) | 334-397 (7.23e-07) | 454-528 (5.19e-35) | |
| LOC100251954 | 5 | 2-81 (3.21e-17) | 78-300 (5.73e-90) | 308-371 (7.82e-07) | 428-502 (5.89e-35) | |
| LOC100247874 | 5 | 29-108 (1.69e-15) | 105-326 (1.95e-85) | 334-456 (4.82e-18) | 486-560 (9.46e-36) | |
| LOC100247881 | 5 | 2-70 (8.16e-12) | 67-289 (1.24e-88) | 297-419 (4.01e-14) | 449-523 (1.21e-33) | |
| LOC100253001 | 5 | 2-70 (2.93e-12) | 67-289 (3.50e-103) | 297-419 (5.21e-12) | 449-523 (1.73e-31) | |
| LOC100259879 | 5 | 1-73 (1.60e-10) | 70-272 (5.91e-80) | 300-428 (1.15e-17) | 458-532 (1.13e-33) | |
| LOC100241049 | 5 | 2-80 (1.84e-14) | 77-29 (7.11e-91) | 307-435 (2.10e-16) | 465-539 (6.90e-33) | |
| LOC100244497 | 5 | 36-114 (8.62e-18) | 111-333 (1.36e-90) | 341-463 (6.75e-17) | 493-566 (6.87e-32) | |
| LOC100247847 | 5 | 28-108 (2.88e-11) | 106-327 (5.91e-80) | 335-455 (1.07e-17) | 485-559 (3.80e-34) | |
| LOC100261541 | 5 | 32-109 (6.88e-14) | 107-329 (7.21e-93) | 337-460 (1.39e-16) | 490-564 (5.75e-33) | |
| LOC100256451 | 5 | 31-98 (3.75e-12) | 96-318 (1.84e-93) | 326-449 (2.60e-09) | 479-553 (2.50e-32) | |
| LOC100266737 | 5 | 42-115 (4.43e-14) | 114-336 (1.23e-82) | 346-414 (3.69e-06) | 455-529 (2.64e-34) | |
| LOC100244417 | 5 | 2-66 (1.01e-10) | 69-291 (1.56e-80) | 301-422 (2.09e-15) | 452-526 (1.49e-32) | |
| LOC100259792 | 5 | 11-70 (1.38e-11) | 68-290 (1.45e-82) | 300-421 (1.47e-14) | 451-525 (2.52e-23) | |
| LOC100263269 | 5 | 11-66 (7.30e-11) | 69-291 (1.27e-90) | 301-421 (7.48e-07) | 451-525 (4.11e-33) | |
| LOC100258131 | 5 | 31-72 (5.74e-07) | 69-291 (1.73e-81) | 299-421 (1.33e-17) | 451-524 (9.38e-32) | |
| LOC100260464 | 5 | 115-190 (7.42e-13) | 190-408 (1.46e-88) | 417-534 (9.44e-13) | 564-638 (2.54e-34) | |
| LOC100253594 | 5 | 100-178 (7.82e-14) | 175-394 (4.31e-80) | 403-523 (2.70e-14) | 535-622 (9.25e-33) | **RNase_HI_RT_Ty1** [1-91 (3.03e-42)] |
| LOC100243364 | 5 | 11-89 (1.37e-15) | 86-306 (4.91e-85 | 314-433 (7.90e-12) | 463-532 (2.52e-32) | |
| LOC100266702 | 5 | 41-109 (2.96e-13) | 106-325 (3.47e-97) | 334-453 (3.80e-06) | 480-557 (2.80e-32) | |
| LOC100241012 | 5 | 2-71 (2.73e-11) | 68-283 (6.16e-86) | 292-409 (4.00e-05) | 436-511 (1.74e-33) | |
| LOC100262117 | 5 | 2-73 (4.57e-13) | 70-268 (1.18e-86) | 297-418 (9.98e-11) | 448-521 (1.77e-33) | |
| LOC100241625 | 5 | 33-110 (3.29e-11) | 109-326 (1.98e-86) | 335-455 (3.67e-17) | 485-559 (1.54e-35) | |
| LOC100259224 | 5 | 32-106 (1.92e-16) | 103-327 (1.40e-89) | 335-457 (9.02e-15) | 486-559 (2.23e-32) | |
| LOC100254106 | 5 | 29-106 (2.29e-13) | 103-325 (3.39e-103) | 335-458 (3.19e-15) | 487-547 (9.56e-23) | |
| LOC100252726 | 5 | 32-108 (1.01e-17) | 109-335 (7.92e-103) | 343-466 (5.14e-16) | 495-569 (7.79e-32) | |
| LOC100250428 | - | 249-335 (3.39e-11) | 332-565 (9.56e-97) | 572-706 (2.65e-21) | 67-152 (6.72e-18);  131-181 (1.40e-03);  736-808 (5.49e-32) | |
| LOC100263349 | 6 | 30-112 (1.33e-11) | 109-328 (5.41e-80) | 335-455 (2.56e-30) | 484-556 (3.39e-32) | |
| LOC100247880 | 6 | 15-96 (9.84e-12) | 95-312 (7.10e-78) | 319-448 (1.40e-26) | 477-545 (2.13e-30) | |
| LOC100242816 | 6 | 38-119 (4.64e-14) | 119-341 (1.09e-88) | 350-475 (4.65e-33) | 504-576 (2.69e-34) | |
| LOC100258212 | 6 | 32-114 (2.23e-18) | 111-335 (4.63e-105) | 336-463 (1.36e-33) | 492-564 (7.65e-34) | |
| LOC100253079 | 6 | 26-109 (5.35e-14) | 106-324 (3.48e-96) | 332-458 (3.45e-28) | 487-559 (3.24e-33) | |
| LOC100247957 | 6 | 59-141 (2.38e-13) | 140-362 (1.85e-88) | 363-490 (7.34e-36) | 519-591 (1.05e-32) | |
| LOC100265217 | 6 | 672-754 (6.82e-16) | 751-987 (2.41e-104) | 995-1121 (9.10e-26) | 1150-1226 (2.57e-35) | **Exostosin** [207-549 (3.98e-71)] |
| LOC100255668 | 7 | 25-117 (2.53e-07) | 116-347 (8.57e-112) | 355-482 (8.44e-28) | 507-586 (2.81e-37) | |
| LOC100260739 | 7 | 27-116 (1.38e-06) | 115-347 (5.91e-91) | 355-477 (3.34e-24) | 507-581 (1.94e-34) | |
| LOC100249001 | 7 | 26-117 (2.15e-07) | 116-374 (5.00e-89) | 356-478 (1.62e-28) | 490-582 (9.95e-33) | |
| LOC100265949 | 7 | 118-207 (6.64e-09) | 206-464 (3.50e-82) | 446-568 (2.14e-29) | 598-672 (2.34e-35) | |
| LOC100257393 | 7 | 25-116 (2.61e-07) | 113-346 (4.36e-78) | 354-476 (3.22e-30) | 453-580 (5.56e-36) | |
| LOC100262514 | 7 | 24-114 (2.68e-08) | 113-344 (6.08e-94) | 352-474 (2.11e-23) | 504-578 (4.00e-35) | |
| LOC100245233 | 8 | 56-127 (4.71e-14) | 130-365 (1.27e-85) | 376-514 (2.29e-13) | 562-633 (6.81e-35) | **DUF1034** [716-811 (8.58e-17)] |
| LOC100242573 | 8 | 83-146 (1.97e-10) | 146-410 (3.47e-91) | 392-527 (5.57e-10) | 579-653 (1.17e-27) | **T7SS_mycosin** [616-694 (1.08e-05)] |
| LOC100243546 | 8 | 59-132 (3.27e-13) | 130-369 (3.65e-98) | 377-512 (2.75e-10) | 569-596 (1.14e-03) | |
| LOC100250404 | 8 | 59-117 (5.53e-06) | 117-359 (2.51e-68) | 368-510 (2.43e-12) | 535-629 (9.11e-36) | **HAP1_N** [790-888 (4.95e-04)] |
| LOC100251210 | 8 | 91-149 (5.79e-06) | 149-391 (5.66e-67) | 400-542 (5.58e-11) | 567-661 (5.54e-35) | **SpoU_methylase** [14-54 (4.35e-09)];  **DUF1034** [740-834 (1.14e-07)] |

Numbers in brackets indicate E-values
