# Supplementary material for: Genome-wide and molecular evolution analysis of the subtilase gene family in Vitis vinifera
Source: BMC Genomics. 2014 Dec 16;15(1):1116. doi: 10.1186/1471-2164-15-1116 (PMC4378017; doi:10.1186/1471-2164-15-1116)
Supplement: Supplementary file 3 — Additional file 3: Table S3: Motif sequences identified by MEME tools. The numbers correspond to the motifs described in Figure 3. (DOC 46 KB) [file 12864_2014_6873_MOESM3_ESM.doc]

**Table S3**. Motif sequences identified by MEME tools.

| **Motif** | **E-value** | **Multilevel consensus sequence** |
| --- | --- | --- |
| 1 | 2.5e-2516 | RSVx[FY]NI[IL]SGTSM[SA]CPH[VA][SA]G[AVI]AA[LY][LIV]K[SA][AF]HP[DT]WSP[AS]AI[KR]SA[LI]MTTAY[TP][ML][DN]N |
| 2 | 1.5e-1348 | FSSRGP[NS]PI[TS]P[DE][IV]LKPD[IV]TAPGV[ND]ILAAW |
| 3 | 2.5e-1339 | G[AS]GH[VI][ND]P[NV][KR]A[ML][DN]PGL[VI]YD[AI]TxxDY[ILV]N[FY]LC |
| 4 | 1.6e-1053 | [GE]SD[VI]I[IV]GV[LI]D[TS]G[IV]WPESESFSD[EK]G[MF]G |
| 5 | 1.9e-1248 | GGC[AY]D[AS]D[IV]LAA[FI]D[DQ]A[IV]ADGVD[IV][LI]S[LI]S[LIV]GG |
| 6 | 2.3e-977 | S[PA]RD[ST][DE]GHGTHTASTAAG[NS]xV |
| 7 | 7.4e-1187 | [YF][FY]ED[PS]IAIG[AS]FHA[MV]K[KN]G[IV][LF][VT][SV]C[SA]AGNSGP |
| 8 | 2.0e-1215 | [ST][VI][ST]NV[AS]PWIL[TS]V[AG]ASTIDR[KSD]FP[TA]KV[VK]LGN[GN] |
| 9 | 1.4e-1170 | x[GN]AS[LY][FL]G[LY][AG]KGTARG[GM][AV]PSAR[IL]AVYK[VA]CW |
| 10 | 8.3e-758 | GF[AVS]A[KR]L[TS]EE[EQ][AL][EK]KL[AS]KM[EDP]GV[VL]SVFP[ND]x |
| 11 | 3.9e-616 | [SF]TCN[RN]K[LI]IGARY[FY]N[KS] |
| 12 | 9.6e-768 | APPG[VL][KS][VI]xVEPSVLSF[KT]SL[GN][EQ]K[KL][ST][FY][KT]VT[VF] |
| 13 | 5.1e-649 | KL[KQ]LHTT[RH][ST][WP]DF[LM]G[LF]PQxVGR |
| 14 | 9.6e-439 | P[PVI]P[SA][KR]WKGTC[EQ] |
| 15 | 6.8e-358 | [DN]PS[LK]VKGK[IV]V[LV]CD |
| 16 | 1.3e-350 | VT[FV]TRT[VL]TNVG |
| 17 | 7.5e-387 | [HF]PLPA[ST]A[VL][SG]AS[DA]GLK[IV]KxYINST[KS |
| 18 | 4.4e-348 | WSDG[KV]HxVRSP |
| 19 | 2.1e-365 | [ST]VW[DN]LNYPS[FI][ATS][VI][SP]FL |
| 20 | 2.2e-276 | KG[EQ][EV]VKxAGG[AV][GA]MIL[AL]NTES[NQ]G[EF][ES][LT][VI]AD[PA] |
| 21 | 1.7e-229 | SASESL[LV]YSYK[RH][SV][FI]N |
| 22 | 2.2e-199 | [GS][LQ]GY[NT]T[TK]Q[IL]RL[IV][TS][GR][DR] |
| 23 | 3.5e-181 | [RK][KQ][VT]Y[IV]V[YH][ML][GD]KLPKGD |
| 24 | 1.3e-179 | PTASILKS[TE][ET]VKD[VE][LS]AP[VFI][VM][AV]S |
| 25 | 2.2e-148 | [GN][KT]M[YL]PL[VI]Y[GA]GDA[GP]N[AT] |

Numbers correspond to the motifs described in **Figure 3**
