# Supplementary material for: Genome-wide and molecular evolution analysis of the subtilase gene family in Vitis vinifera
Source: BMC Genomics. 2014 Dec 16;15(1):1116. doi: 10.1186/1471-2164-15-1116 (PMC4378017; doi:10.1186/1471-2164-15-1116)
Supplement: Supplementary file 4 — Additional file 4: Table S4: Primers used for quantitative real-time qRT-PCR of Vitis vinifera. (DOC 110 KB) [file 12864_2014_6873_MOESM4_ESM.doc]

**Table S4**. Primers of real-time quantitative PCR (qRT-PCR) used in this study*.*

| Prime name | Sequence |
| --- | --- |
| LOC100241012- F | CCCTGGATTGTGATGTACCCT |
| LOC100241012 -R | TGCAAATTCTTGTTCCCCTAAA |
| LOC100241049-F | GGCAGTGGTAGGGCATCAA |
| LOC100241049-R | TGTGGAGCTGTTTCTTTTCGTT |
| LOC100241625-F | ACAACAAGCCCACGACTCAAG |
| LOC100241625-R | CCAGCGAAGCAGACTCCATT |
| LOC100242388-F | TTGGGGCAATGGAAAAGG |
| LOC100242388-R | GGCTAACGAAGGCAGGGA |
| LOC100242573-F | GGCATGGGTCCAAGAAGG |
| LOC100242573-R | CAGATGGGAAATCAGGGGTC |
| LOC100242816-F | CAGCCAGACTGACGGAGGA |
| LOC100242816-R | ATCGGGTGAATGCGTTGTT |
| LOC100243364-F | TTCTGCTCCTGTAGTGGCTCC |
| LOC100243364-R | TCAAGTATGGCTGCGTTTGG |
| LOC100243546-F | GCCCCCATTGTGAGCAGAT |
| LOC100243546-R | GCCCTGGTGCCAAAATATCA |
| LOC100243634-F | GCTTAGTCGGCGATGCTCA |
| LOC100243634-R | TGGCTTGATTCCTATCACAGTTC |
| LOC100243797-F | GAAGTTTGTCGTTGTCCAGTGAT |
| LOC100243797-R | TGGCGGACCGAATAGCA |
| LOC100243842-F | TCTCCAACATCAACTACCCATCCA |
| LOC100243842-R | TGCCCTCTCTAAACCTTCCACAA |
| LOC100244417-F | TCATCCCTCCACGCTAACATACTT |
| LOC100244417-R | CGACTCCTCCTCGGTCAACTT |
| LOC100244497-F | GGACGGTGATGGCGGATAG |
| LOC100244497-R | GACCTCAGTGCTCTTCAATATGGA |
| LOC100247847-F | GATGCTGGCGAGACGGATT |
| LOC100247847-R | CCTTGTGAAGTTGCGAGTAATGC |
| LOC100247874-F | GGACGGTTACTAATGTTGGCTCTC |
| LOC100247874-R | TGATGGGCTGTTGCGAAATCTTA |
| LOC100247880-F | AGTCCTGCTTTCCTGGGTTTG |
| LOC100247880-R | GTGGCATTCCTTCGTCGCTAA |
| LOC100248833-F | TGTCATCGCCATTGCTTCTCTT |
| LOC100248833-R | TGTCCCACCATCATTCCCAGTA |
| LOC100248908-F | CCAGGAACTCTAAGCACACTCAA |
| LOC100248908-R | TACAGGGAACGCAAAGGCAAT |
| LOC100249001-F | TGGAACTCACACCGCATCAAC |
| LOC100249001-R | AGCACAACCACCAGCATAACAA |
| LOC100250276-F | GCACACCCTCCACACTACAAG |
| LOC100250276-R | GCCATACACCATTATCCACCATCC |
| LOC100250404-F | ATCAATCCATCCCGTGCCATC |
| LOC100250404-R | CGCCAACTGCTCGTCTCAT |
| LOC100250428-F | AGTTGGCTGGTTCTGATGATTCTT |
| LOC100250428-R | CGTTCCTGTGCTTGCTGATGA |
| LOC100251409-F | GCACACCAGATTCACACCACT |
| LOC100251409-R | ATCACATCATCGGCGTAATCAGAG |
| LOC100251507-F | AAGGTTATGCTGCTTATGCTGGT |
| LOC100251507-R | CGCTGAGTTGAAGGAGGAGTTG |
| LOC100252726-F | TGTCACCAGCGAGGAAGGA |
| LOC100252726-R | GCTTGAGGATGTTGGGACTGAG |
| LOC100253001-F | GGTCTAGTGTACGATGCCAATGAG |
| LOC100253001-R | TTGCCGCAGAGCCTTAGC |
| LOC100253079-F | CAGCCAGACTAACCGAAGATGAAG |
| LOC100253079-R | CAGCCAGACTAACCGAAGATGAAG |
| LOC100253196-F | GAACTCGCTGTGCCTGGA |
| LOC100253196-R | CAACTGCTGGAAGAAGGTGAGAA |
| LOC100254106-F | GACACAGTCGGACAAGCCATT |
| LOC100254106-R | ACGCCAGGTGCTACAACATC |
| LOC100254813-F | CAGCAAGATGGAAAGGAGAGTGT |
| LOC100254813-R | GCAGCGGTGGAAGAAGTATGT |
| LOC100254828-F | TCTTGCTAACCGTCTGTCCACTA |
| LOC100254828-R | TGTACCTACTTCAGTTGCTCCTCT |
| LOC100255612-F | CCAGACCACAGAGCGATTACAT |
| LOC100255612-R | GCAATGGCGGAGACAATAGGA |
| LOC100255614-F | AGTTGGCTGGTTCTGATGATTCTT |
| LOC100255614-R | AGTTGGCTGGTTCTGATGATTCTT |
| LOC100255668-F | TGCCACTCCAACTGCTTCA |
| LOC100255668-R | TCTTCTTCACCTCCTTCTTCTTCA |
| LOC100256451-F | CCAGGACGGTCACGAATG |
| LOC100256451-R | TAAGGACAGGTGGCTCAACT |
| LOC100256591-F | TTTATGGTGTGGGCAAAGGAACT |
| LOC100256591-R | GATGTCAGAATCGTAGCAGGATGG |
| LOC100257393-F | TCAAGGAGGAGGCGAAGAAGA |
| LOC100257393-R | GTGAGTGTGTGGTGTGGAGAC |
| LOC100257482-F | AGACTGACACTGCTGACTACTTG |
| LOC100257482-R | GTTCTGCTTACCTTCTTGCTCTCA |
| LOC100258131-F | TTGGCAGTAGAGCATCGGAATATC |
| LOC100258131-R | TGACCTTGTCGTGTGGAGTTG |
| LOC100258241-F | GCTACAACAACTCTGCCATCTCT |
| LOC100258241-R | ACACCAATAGGAGGCTGAATCAC |
| LOC100259937-F | TATGACTTGACCACCACTGACTA |
| LOC100259937-R | CACTGTTACCTTGCCTGAGAG |
| LOC100260464-F | AACCGAGTTTCCTCTTGTGTATGG |
| LOC100260464-R | TCTCTGTCTCCTGTGATGCTTCT |
| LOC100260528-F | TCTGTGAAGGATGAGTATGGTCTA |
| LOC100260528-R | GTGGATAGCAGTGGTGTTGTAG |
| LOC100260681-F | GGTCCTCCTCAACAATACTAACAC |
| LOC100260681-R | GTAGAAGGCAGCACGGTAAC |
| LOC100260739-F | CGTTGCTGGTGGTCTTCAT |
| LOC100260739-R | TGCTTCTTCTCCTCCATCTTCT |
| LOC100262117-F | CCTCAACAGCAATTCTCACCTT |
| LOC100262117-R | AACAGACACCACTTCCTCCTTA |
| LOC100264034-F | ACGACGGAGTAGATGTGTTATCA |
| LOC100264034-R | GGAATGAACCAATGAGGATGCTAT |
| LOC100265129-F | GACCTTAGCGACAACCAGAAG |
| LOC100265129-R | GCATTATAGCCAATAGAGCACAGA |
| LOC100265217-F | GAGAGCATTGAGAACTGTTGAGAA |
| LOC100265217-R | ACTCCTTCAGCATATCCTCGTTA |
| LOC100265607-F | GTTGGATATGGCGGAGAAGG |
| LOC100265607-R | TAGATAGGAGTTGGTGATGGTGTT |
| LOC100266737-F | GTCAAGTGTCTGCGTCATCC |
| LOC100266737-R | CTCCTCCTCCGTCAACCTT |
| LOC100267603-F | AGAAGCAAGGAACTATGTGAAGAC |
| LOC100267603-R | GGTTGAAGTAGCAGAACACGATT |
| Grape tubulin-F | GATGTGTGCTGCTGATCCAC |
| Grape tubulin-R | TCACCCTCCTGAACATCTCC |
